# Blood-Pressure-Monitoring Smartphone Applications: Ushering in a New Era of Wearable Cardiac Devices? Comment on Vischer et al. Comparability of a Blood-Pressure-Monitoring Smartphone Application with Conventional Measurements—A Pilot Study. *Diagnostics* 2022, *12*, 749

**DOI:** 10.3390/diagnostics13020290

**Published:** 2023-01-12

**Authors:** Sara Hungerford, Nicole Bart

**Affiliations:** 1 Department of Cardiology, Royal North Shore Public Hospital, Sydney, NSW 2065, Australia; 2 St Vincent’s Hospital Clinical School, Faculty of Medicine, University of New South Wales, Sydney, NSW 2010, Australia; 3 Department of Cardiology, St Vincent’s Hospital, Sydney, NSW 2010, Australia

Systemic arterial hypertension in adults is generally defined as a systolic blood pressure (SBP) of >140 mmHg and/or a diastolic blood pressure (DBP) of >90 mmHg [1] and represents a major challenge worldwide owing to a rapid year-by-year increase in prevalence [2,3,4]. There are well-recognised challenges in blood pressure (BP) measurement by physicians in the clinical setting [5]. As such, methods to evaluate systemic hypertension have evolved considerably over the last two decades, moving away from a conventional office-based approach towards integrated, automated techniques that can be performed unattended. The emergence of enhanced smartphone technologies shows great promise in this space.

In this issue of Diagnostics, “Comparability of a Blood-Pressure-Monitoring Smartphone Application with Conventional Measurements” [6], Vischer et al. compare a smartphone application-based algorithm (termed AppBP) with an office-based BP monitoring approach (termed CuffBP). This study enrolled consecutive patients with an indication for ambulatory BP monitoring. Smartphone app technology (RIVA digital) was custom designed to acquire the pulse wave in the fingers’ arterial bed using the smartphone’s camera and then estimate BP based on an analysis of photoplethysmographic (PPG) waveforms [6].

Measurements were alternatingly taken with the AppBP or CuffBP on two consecutive days. Four measurements per day resulted in four hundred valid CuffBP values. After calibration, each smartphone BP recording was compared to the mean of the previous or following CuffBP recording. A total of 50 patients were analysed (of which 48% were female). Thirty-eight participants (76%) had a known history of hypertension, and thirty (60%) were receiving antihypertensive treatment at the time of the study [6]. The systolic BP readings ranged from 89 to 202 mmHg for all 400 CuffBP values, with a mean of 126 ± 16 mmHg. The diastolic BP ranged from 48 to 96 mmHg, with a mean of 79 ± 8.7 mmHg. A total of 4% of the systolic CuffBP values were ≤100 mmHg, 15% were ≥140 mmHg, and 3% were ≥160 mmHg [1]. Additionally, 3% of the diastolic CuffBP values were ≤60 mmHg, 28% were ≥85 mmHg, but none were ≥100 mmHg [6].

Notably, the post-recording quality threshold was not met in 225 AppBP-based measurements, which had to be excluded. This left 175 uncalibrated values and 83 calibrated values. Importantly, the uncalibrated systolic AppBP values were significantly lower than the CuffBP values (*p* < 0.0005); as a result, only the AppBP sets with a valid first AppBP measurement were included for subsequent analysis. Reassuringly, the calibrated systolic AppBP values (median 127.6 [IQR 113.8–135.3] mmHg) did not significantly differ from the corresponding systolic CuffBP values (median 125.0 [IQR 115.0–132.0] mmHg, *p*-value 0.234, z-value 1.190); however, the calibrated diastolic AppBP values (median 79.3 (IQR 74.0–83.6) mmHg) were significantly higher than the corresponding diastolic CuffBP values (78.5 [IQR 72.0–82.5] mmHg, *p*-value 0.041, z-value 2.039) [6].

Overall, *Vischer* et al. found that an AppBP assessment alone without calibration was insufficient to meet the quality threshold in more than 50% of the participants, as there were significant differences between the AppBP and CuffBP values. After calibration, however, the agreement between AppBP and CuffBP values was improved (especially for systolic BP measurement) [6]. The author(s) concluded that the PPG-based algorithm with single-point calibration was of sufficient quality to meet established international standards [7,8].

One limitation is that in both arms of the study, patients were asked to attend the office. White-coat hypertension is a well-documented phenomenon, and the digital era has facilitated the uptake of home-based strategies [9]. Home and ambulatory monitoring have proven to be better prognostically in predicted end-organ damage [10]. Moreover, telemedicine has been shown to reduce SBP and DBP when compared to usual care [11]. In addition, the self-monitoring of BP has been shown to increase patient autonomy and compliance with therapy [11].

Vischer et al. demonstrate that an AppBP model that utilises PPG technology can record BP with sufficient accuracy. The authors should be commended for their innovative work in bringing together an array of technologies. In so doing, the authors have elegantly demonstrated a PPG model that may be applied in future population studies where the diagnosis of hypertension is in question.

The diagnosis of hypertension can be, at times, challenging and imprecise in the clinical setting. Advanced smartphone technology systems are indispensable tools in this regard. Though this is a pilot study with a relatively small dataset, it is the first to demonstrate that a PPG-based app approach is viable. Undoubtedly, the quality of data was improved with the addition of calibration, and the high PPG data rejection rates are a clear indication that a post-recording check is also crucial. Nevertheless, this study provides insight into the prognostic value of integrating PPG-based smartphone applications into the hypertension diagnostics space [6]. This technology can only be strengthened with home-based monitoring, continuous monitoring to identify stressors, and the potential incorporation of artificial intelligence.

Overall, Vischer et al. demonstrate that an AppBP model that utilises PPG technology can record BP with sufficient accuracy. The author(s) should be commended for their innovative work in bringing together an array of technologies. In so doing, the author(s) have elegantly demonstrated a PPG model that may be applied in future population studies where the diagnosis of hypertension is in question. Future prospective studies of this model with other systems are warranted.
